# PTH-driven modulation of platelet activity via the NOX2 pathway in postsurgical hypoparathyroidism

**DOI:** 10.1016/j.redox.2025.103896

**Published:** 2025-10-14

**Authors:** Alessandra D'Amico, Gaia Tabacco, Cristina Nocella, Anda Mihaela Naciu, Annunziata Nusca, Francesco Piccirillo, Michele Mattia Viscusi, Federico Bernardini, Valentina Valenti, Angela Leonardo, Sebastiano Sciarretta, Ernesto Greco, Gianmarco Sarto, Beatrice Simeone, Luca D'Ambrosio, Giacomo Frati, Athanasios D. Anastasilakis, Francesco Grigioni, Nicola Napoli, Maurizio Forte, Andrea Palermo, Roberto Carnevale

**Affiliations:** a IRCCS Neuromed, Pozzilli, Italy; b Unit of Metabolic bone and thyroid disorders, Fondazione Policlinico Universitario Campus Bio-Medico, Rome, Italy; c Department for the Promotion of Human Science and Quality of Life, San Raffaele Open University, Rome, Italy; d Department of Clinical Internal, Anesthesiology and Cardiovascular Sciences, Sapienza University of Rome, 00161, Rome, Italy; e Unit of Cardiac Sciences, Department of Medicine, Campus Bio-Medico University of Rome, Cardiology Unit, Fondazione Policlinico Universitario Campus Bio-Medico of Rome, Rome, Italy; f Department of Medical and Surgical Sciences and Biotechnologies, Sapienza University of Rome, Latina, Italy; g Maria Cecilia Hospital, GVM Care & Research, Cotignola, Ravenna, Italy; h Department of Health and Life Sciences, European University of Rome, Via degli Aldobrandeschi 190, 00163, Rome, Italy; i Cardiology Division, ICOT Istituto 'Marco Pasquali' University Hospital, Via Franco Faggiana 1668, Latina, 04100, Italy; j Department of Endocrinology, 424 General Military Hospital, Thessaloniki, Greece; k Unit of Endocrinology and Diabetes, Campus Bio-Medico University, Rome, Italy

**Keywords:** PTH, Hypoparathyroidism, NOX2, Oxidative stress, Redox signalling, Platelet activation

## Abstract

**Objective:**

Postsurgical chronic hypoparathyroidism (HypoPT) has been linked to an increased cardiovascular risk, but the underlying pathophysiological mechanisms remain incompletely understood. Emerging evidence suggests a potential direct role of parathyroid hormone (PTH) in modulating platelet function and oxidative stress, both contributors to atherothrombosis.

Our study aimed to investigate the impact of PTH on platelet function and activation, with a particular focus on NOX2-mediated platelet activation in patients with HypoPT.

**Methods:**

We conducted a cross-sectional study involving 24 patients with HypoPT and 40 age- and sex-matched healthy controls. Clinical, biochemical, and platelet function parameters were assessed. In a subgroup of five HypoPT patients, changes were evaluated after 24 months of PTH (1–34) therapy. Platelet aggregation, oxidative stress biomarkers (sNOX2-dp, H_2_O_2_, 8-OHdG), and thrombus formation (T-TAS) were measured. The in vitro effect of PTH (1–34) was tested on isolated platelets.

**Results:**

Patients with HypoPT exhibited enhanced platelet activation, increased oxidative stress, and accelerated thrombus formation compared to controls. Enhanced platelet activation and increased oxidative stress observed in HypoPT were further amplified in HypoPT subjects treated with PTH (1–34). In vitro, PTH (1–34) increased oxidative stress and platelet aggregation only in platelets from HypoPT patients, through a specific signaling pathway involving PTH1R activation, intracellular calcium release, protein kinase C (PKC) activation and NOX2-dependent ROS generation.

**Conclusion:**

HypoPT is associated with heightened platelet reactivity and thrombotic risk. PTH therapy may exacerbate these alterations through a defined molecular mechanism. These findings highlight the need for careful cardiovascular monitoring in HypoPT patients, particularly those receiving PTH analogues.

## Highlights

• Hypoparathyroidism is linked to enhanced oxidative stress and platelet activation.
• Recombinant PTH (1–34) exacerbates platelet activation and thrombus formation in HypoPT.
• PTH signaling involves PTH1R–calcium–PKC–NOX2 axis in platelets.
• In vitro, PTH increases oxidative stress and platelet aggregation in HypoPT but not in control platelets.
• Findings support cardiovascular risk evaluation in HypoPT patients on PTH analogues.

## Graphical abstract

**Figure:**
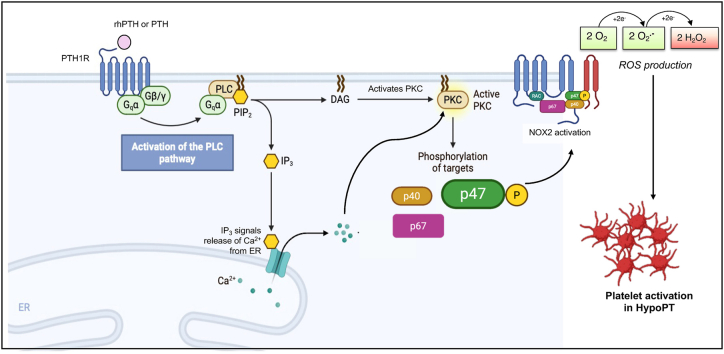

## Non-standard abbreviations and acronyms

PHPT -: primary hyperparathyroidism
HypoPT -: hypoparathyroidism
PTH -: parathyroid hormone
rhPTH –: recombinant human parathyroid hormone
PTHrP -: parathyroid hormone related protein
cAMP -: cyclic adenosine 3′,5′-monophosphate
PKC -: protein kinase C
MAPK -: mitogen-activated protein kinase
ERK: extracellular ligand-regulated kinases
NOX2 -: nicotinamide adenine dinucleotide phosphate oxidase 2
8-OHdG -: 8-hydroxy-2′-deoxyguanosine
25(OH)D -: 25-hydroxy vitamin D
H_2_O_2_ -: hydrogen peroxide
PRP -: platelet-rich plasma
PPP –: platelet-poor plasma
CD40L -: cluster of differentiation 40 ligand
BAPA -: benzylsulfonyl-D-Arg-Pro-4-amidinobenzylamide
vWF -: von Willebrand factor
DAG -: diacylglycerol
ICAM-1 -: intercellular adhesion molecule-1
VCAM-1 -: vascular cell adhesion molecule-1

## 1 Introduction

Parathyroid disorders, particularly primary hyperparathyroidism (PHPT), have been consistently associated with an increased risk of cardiovascular morbidity and mortality.

This increased risk has been linked not only to classical cardiovascular risk factors, such as hypertension and dyslipidemia, but also to non-traditional mechanisms including endothelial dysfunction, vascular calcification, and enhanced oxidative stress [[1], [2], [3]].

Several studies have shown that patients with PHPT exhibit elevated markers of oxidative stress and reduced antioxidant capacity, alterations that may improve after parathyroidectomy [4]. Elevated parathyroid hormone (PTH) levels have also been implicated in mitochondrial calcium overload, leading to necrotic cell death and further propagation of oxidative damage [5].

In addition to PHPT, hypoparathyroidism (HypoPT), a condition marked by chronic PTH deficiency, has been associated with increased all-cause mortality, and a higher incidence of heart failure, myocardial infarction, arrhythmias (including atrial fibrillation), valvular heart disease, and peripheral vascular disease compared to matched controls [[6], [7], [8]].

Taken together, these findings suggest that parathyroid disorders may exert direct effects on vascular biology and platelet function, thereby contributing to the observed excess cardiovascular burden.

Despite this well-established association, the underlying molecular mechanisms by which parathyroid dysfunction contributes to cardiovascular disease remain not completely understood, especially concerning its impact on endothelial dysfunction and platelet activation, both key pathophysiological drivers of atherothrombosis [9]. Previous studies have indicated that parathyroid dysfunction may adversely affect the cardiovascular system through disruptions in calcium-phosphate homeostasis, such as hypercalcemia, hypocalcemia, hyperphosphatemia, and elevated calcium-phosphate product [1,7].

Emerging evidence suggests that parathyroid hormone (PTH) and calcium-phosphate imbalance may trigger a cascade of detrimental effects on the vascular endothelium, including increased oxidative stress, upregulation of pro-inflammatory cytokines, and dysregulation of nitric oxide (NO) signaling pathways [5]. These alterations can impair endothelial barrier function, promote vascular smooth muscle proliferation, and facilitate leukocyte adhesion, collectively creating a pro-atherogenic environment [10].

Moreover, parathyroid hormone (PTH) may exert direct cardiovascular effects via binding to its cognate receptors, PTH1R and PTH2R, which activate intracellular signaling cascades including cyclic adenosine monophosphate (cAMP), protein kinase C (PKC), and mitogen-activated protein kinase (MAPK) pathways in cardiomyocytes and endothelial cells [11,12].

Notably, platelets also express PTH1R, mediating pro-aggregatory responses to both PTH and PTH-related protein (PTHrP), via extracellular signal-regulated kinases 1 and 2 (ERK1/2) activation [13]. Additionally, disruption of PTH1R phosphorylation prolongs ERK1/2 activation and enhances c-fos expression, indicating a sustained pro-aggregatory state in platelets [14].

Recently, we reported that patients with both PHPT and HypoPT exhibit increased markers of atherothrombotic risk, driven by alterations in endothelial and platelet function [15].

However, in the context of HypoPT, there is still a limited amount of preclinical and clinical data available on platelet function. Finally, patients with HypoPT who are not adequately managed with conventional therapy may be considered for replacement treatment with PTH analogues. Given the potential role of PTH in modulating cardiovascular physiology, a critical gap remains in knowledge regarding the molecular effects of PTH on platelet activation, endothelial dysfunction, and oxidative stress in individuals with chronic HypoPT. Accordingly, the present study aimed to investigate the impact of PTH on platelet function and activation, with a particular focus on NOX2-mediated platelet activation in patients with HypoPT.

## 2 Materials and methods

### 2.1 Study design

This cross-sectional, single-center study was carried out between March 2021 and March 2024 at the Bone Outpatient Clinic of the Metabolic Bone and Thyroid Disorders Unit, Fondazione Policlinico Universitario Campus Bio-Medico, Rome. The protocol of this study adhered to the principles of the Declaration of Helsinki and the International Conference on Harmonization Good Clinical Practice guidelines, with approval granted by the Ethics Committee of 10.13039/501100003765Campus Bio-Medico University (89/20 10.13039/100030240PAR ComEt CBM, November 27, 2020). All participants provided informed consent for the use of their pseudoanonymized data in the analysis.

HypoPT subjects were consecutively enrolled in the study. HypoPT was defined as hypocalcemia accompanied by low or inappropriately normal PTH levels persisting for at least 12 months following surgery [16]. Exclusion criteria included any condition potentially affecting bone or calcium metabolism, malabsorption syndromes, other known disorders impacting bone health, and the use of medications influencing bone and calcium metabolism. Additionally, individuals with a history of cardiovascular disease, symptoms suggestive of atherosclerotic cardiovascular disease, or classified as high or very high cardiovascular risk according to the SCORE2 system were excluded [17]. Patients with active malignancies, except for low-risk, well-differentiated thyroid cancer, were also not considered eligible. Age- and sex-matched healthy controls, with normal calcium, phosphate, and PTH levels, were consecutively recruited from the endocrinology outpatient clinic, where they had been referred for unrelated conditions (euthyroid thyroid nodules). The same exclusion criteria were applied to the control group. The clinical profile of the entire study population was assessed through medical history collection, along with a review of existing clinical, laboratory, and imaging records. Additionally, all subjects underwent a physical examination. All study participants also underwent peripheral blood sampling.

A small subgroup of five patients with HypoPT who initiated treatment with PTH (1–34) at a dose of 40 μg/day during the study period was also included in a longitudinal follow-up phase. These patients underwent a second blood sample collection 24 months after starting PTH (1–34) therapy.

### 2.2 Blood sample collection

After an 8-h fast, venous peripheral blood samples were collected from the participants in the morning. Blood samples were drawn from the antecubital vein and transferred into vacutainer sterile tubes (Franklin Lakes, NJ, USA) for different analyses. Serum total calcium (normal, 8.4–10.2 mg/dL) and albumin were measured using automated methods, and calcium values were corrected for albumin concentration [18]. Serum thyroid-stimulating hormone, blood glucose levels, serum phosphate, 25(OH)D and creatinine were also measured by automated techniques. Intact PTH was measured by an immunochemiluminometric assay using the automatic analyzer Modular E170 (Roche Diagnostics, Indianapolis, Ind, USA). Normal serum iPTH levels ranged between 13 and 85 pg/mL. Serum samples were obtained after centrifugation at 300×*g* for 10 min at room temperature (RT) from anticoagulated blood samples. The supernatants were collected and stored at −80 °C for analyses. Plasma samples were obtained after centrifugation (180×*g* for 15 min at RT) of whole blood samples collected into vacutainer tubes with sodium citrate buffer (1:9 v/v ratio). After the centrifugation, 75 % of the supernatant was collected and stored at −80 °C until further analyses.

### 2.3 Evaluation of oxidative stress biomarkers

The activation of the NOX2 enzyme was assessed both in the serum samples and the supernatants, through an ELISA test according to Carnevale et al. [19], which measured the levels of soluble fragments released into the serum after NOX2 activation [[20], [21], [22], [23], [24], [25], [26]]. Specifically, the NOX2 soluble-derived peptide (sNOX2-dp) was detected using an antibody targeting the amino acid sequence corresponding to the extracellular portion of NOX2 released upon enzyme activation. Results were expressed in pg/mL, intra-assay and inter-assay coefficients of variation were 8.95 % and 9.01 % respectively. The hydrogen peroxide (H_2_O_2_) was detected in the serum samples and supernatants with a colorimetric Detection kit (Arbor Assays Hydrogen Peroxide colorimetric activity kit, Cat #K034–H1). The values were expressed in μM; the intra- and inter-assay coefficients of variation were 4.2 % and 3.4 %, respectively. Serum 8-hydroxy-2′-deoxyguanosine (8-OHdG) levels were measured using a commercially available sandwich enzyme-linked immunosorbent assay (ELISA) kit (Cat #ab285254, Abcam, Cambridge, UK), following the manufacturer's instructions. Concentrations were determined using a standard curve and expressed in ng/mL. A total of 40 healthy controls and 24 patients were analyzed.

P47^phox^ expression was assessed in platelets of HS and HypoPT subjects through western blotting. Platelet samples were lysed in RIPA Buffer (5 mM EDTA, 0.15 mol NaCl, 0.1 mol Tris pH 8.0, 1 % Triton) supplemented with protease and phosphatase inhibitors coktail (10 μg/mL; Thermo Fisher Scientific, Waltham, Massachusetts, USA) and sonicated three times (for 10 s and 70 % amplitude). Lysates were incubated on ice for 30 min and centrifuged at 10,000×*g* for 20 min at 4 °C. Supernatants were collected and protein concentration was determined using the DC protein assay (DC Protein Assay Kit I, Biorad Cat #5000111EDU).

Equal amounts of protein (30 μg) were. mixed with 4x Laemmli sample buffer, (BioRad, Cat #1610747) containing 20 % of 2-mercaptoethanol denatured at 95 °C for 5 min, and separated on 4–20 % Mini-PROTEAN TGX Precast Protein Gels (BioRad Cat #4561095). Proteins were then transferred onto nitrocellulose membranes (0.45 μm pore size, Trans-Blot Turbo Mini Nitrocellulose, Bio-Rad). Membranes were blocked for 1 h at room temperature with 5 % BSA in Tris-buffered saline containing 0.1 % Tween-20 (TBS-T) and then incubated overnight at 4 °C with a primary antibody against p47^phox^ (Cell Signaling Technology, Cat #4301S) and phospho-P47^phox^ (AMSBIO, Cat #A01586S370); followed by incubation with HRP-conjugated secondary anti-mouse (BioRad, Cat #1706516) or anti-rabbit (BioRad, Cat #1706515) antibodies. Protein expressions were detected by enhanced chemiluminescence substrate (Clarity Max Western ECL Substrate, BioRad, Cat #1705062). Densitometric analysis of the bands was performed using Image Lab software. The results were expressed in arbitrary units (A.U.).

### 2.4 Platelet preparation and aggregation

To obtain Platelet-Rich Plasma (PRP), blood samples drawn in 3,2 % sodium citrate tubes were centrifuged at 180×*g* for 15 min at RT. The 75 % of the supernatant was collected and divided into different aliquots. Platelet-poor plasma (PPP) was obtained after centrifugation at 3000×*g* for 5 min of PRP. Platelet aggregation (PA) was induced in the PRP (2 × 10^5^ platelets/μL) samples in the presence of subthreshold concentration of collagen (BioData, Cat #101562) and measured by an eight-channel aggregometer (model PAP-8E, BioData, Horsham, PA, USA) provided with silicon-coated glass cuvettes maintained at 37 °C under stirring continuous conditions (1200 rpm). Collagen induces the formation of aggregates, which in turn cause an increase in the light transmission through the plasma sample (from 0 % for the optical density of autologous PPP to 100 % for the maximum optical density of PRP), registered by a photometer. Then, the PRP analyzed samples were transferred into conical tubes containing 10 % (v/v) ACD buffer (2.5 % sodium citrate; 1.5 % citric acid; 2.0 % dextrose) and centrifuged at 3000 rpm for 3 min at RT. Platelet pellets and supernatants were stored at −20 °C for the following analysis.

### 2.5 P-selectin levels

P-selectin soluble levels (sP-selectin) were measured in the plasma and supernatants through a commercial ELISA kit (Cusabio Cat #CSB-E04708h, RRID: AB_3668981). The values were expressed in ng/mL; the intra- and inter-assay coefficients of variation are <8 % and <10 %, respectively.

### 2.6 CD40L levels

Cluster of Differentiation 40 Ligand soluble levels (sCD40L) were evaluated in the plasma and supernatant samples with a commercial ELISA kit (FineTest Cat #EH0086, RRID: AB_3668948). Values were expressed as pg/mL; both intra- and inter-assay coefficients of variation were within 10 %.

### 2.7 Ex vivo total thrombus-formation analysis system (T-TAS)

Peripheral blood samples were drawn from healthy subjects (HS) and HypoPT subjects in vacutainer sterile tubes provided with Benzylsulfonyl-D-Arg-Pro-4-amidinobenzylamide (BAPA) anticoagulant. For the T-TAS analyses, 320 μL of anticoagulated blood was transferred into a sample holder test tube and injected into microchips with biofunctionalized channels (PL-CHIP, made of collagen-coated microcapillary network). To evaluate the hemostatic functionality and the thrombotic characteristics of the blood in conditions that simulate real vascular flow, the test was run at basal conditions in samples from 18 HypoPT subjects on conventional therapy and 18 HS. For this analysis we used 18 HS from a previously characterized reference cohort. Furthermore, in HypoPT blood samples, 49 pg/mL of rhPTH (1–34) (ProSpec; Cat #HOR-290) was added for additional analysis. During the analysis, the device monitored the thrombus formation by recording the increasing pressure in the microchannels. The thrombus formation was registered by the occlusion time (OT), which represents the time required for the pressure inside the capillaries to reach 60 kPa and the area under the curve (AUC) of the pressure-time graph, which is related to the global amount of thrombus formed over the analysis time.

### 2.8 Von Willebrand factor

Von Willebrand factor (vWF) antigen levels were evaluated in the plasma and supernatants with a commercial ELISA KIT (FineTest Cat #EH1064, RRID: AB_3668983). Values were expressed in ng/mL, intra-assay CV<8 %, and inter-assay CV<10 %.

### 2.9 DAG levels

Diacylglycerol (DAG) levels were evaluated in the serum samples with a commercial ELISA KIT (Abbexa, Cat #abx258320). Values were expressed in ng/mL and the intra- and inter-assay coefficients of variation were <10 % and <12 % respectively.

### 2.10 Intracellular calcium levels

Basal intracellular calcium levels were measured with a Fluo-4 Direct Calcium Assay Kit (Thermo Fisher Scientific, Cat #F10471). Platelets were resuspended in HBSS buffer supplemented with 20 mM HEPES and incubated with the Fluo-4 Direct reagent for 30 min at 37 °C, followed by an additional 30-min equilibration at room temperature.

Fluorescence was then measured using a Varioskan LUX multimode reader (Thermo Fisher Scientific), set to an excitation wavelength of 494 nm and an emission wavelength of 516 nm. The obtained fluorescence readings were corrected for background fluorescence; values were expressed in arbitrary units (A.U.)

### 2.11 cAMP levels

Cyclic adenosine monophosphate (cAMP) levels were evaluated in the serum samples with a commercial ELISA KIT (Elabscience, Cat #E-EL-0056-96T) according to the manufacturer's instructions. Values were expressed in ng/mL, the intra-assay and inter-assay coefficients of variation were <10 %.

### 2.12 In-vitro experiments

Platelet aggregation was monitored through light transmission aggregometry in the PRP samples (250 μL) from HS and HypoPT subjects after a pre-incubation (10’ at 37 °C) with growing concentrations (15–49–85 pg/mL) of rhPTH (1–34), and collagen subthreshold stimulus as agonist of platelet aggregation.

Platelet-rich plasma samples of HypoPT patients were also pre-incubated (10 min at 37 °C) with different inhibitors: Parathyroid Hormone (7–34) Human Recombinant (100 nM; Prospec; Cat # HOR-266) as PTH/PTHrP receptor antagonist, NOX2-ds-tat (10 μM; Anaspec, Fremont, CA, USA) as NOX2 inhibitor and EDTA (1,04 mM; Cat #15825388, Fischer Scientific) as calcium chelator and then stimulated with collagen subthreshold. After the monitoring of platelet aggregation, the treated samples were transferred into conical tubes, added with ACD (10 % v/v) and centrifuged to separate pellets and supernatants. The two fractions were stored for the Western blot analysis and the soluble markers evaluation.

Western blotting was performed as described above. Membranes were incubated overnight at 4 °C with the following primary antibodies: PTH1R (Abcam, Cat #AB75150), PKC (Cell Signaling, Cat #46809S), phospho-PKC (Cell Signaling, Cat #9371S), p47^phox^ (Cell Signaling, Cat #4301S), phospho-p47^phox^ (AMSBIO, Cat #A01586S370), and vinculin (Cell Signaling, Cat #13901S) as a loading control. Antibodies were diluted 1:1000 in EveryBlot Blocking Buffer (BioRad, Cat #12010020). After washing, membranes were incubated for 1 h at room temperature with HRP-conjugated anti-mouse (BioRad, Cat #1706516) or anti-rabbit (BioRad, Cat #1706515) secondary antibodies (1:3000). Protein signals were detected using enhanced chemiluminescence substrate and quantified by densitometric analysis with Image Lab software. Data were expressed in arbitrary units (A.U.) and represented as the mean of three independent experiments.

### 2.13 Statistical analysis

The characteristics of the study participants were reported using descriptive statistics by groups defined according to the diagnosis of HypoPT or control group. Data are expressed as frequencies and percentages for categorical variables and mean ± standard deviation or median [Q1, Q3] for continuous variables. The Shapiro-Wilk test was used to identify deviations from the normal distribution. Differences between the two groups in parametric and non-parametric continuous variables were tested with the student t-test and Mann–Whitney test, respectively. A two-tailed p-value <0.05 was considered statistically significant. Analysis of variance (ANOVA) or the Kruskal-Wallis test was used to compare continuous variables between groups, whether normal or non-normal. A two-tailed p-value <0.05 was considered statistically significant. All statistical analysis was performed using GraphPad Prism 10.

## 3 Results

### 3.1 Clinical and biochemical features

The baseline characteristics of the study population are summarized in Table 1. The study enrolled 24 patients with HypoPT and 40 healthy controls matched for age and sex. The majority of participants were female, with 96 % (n = 23) in the HypoPT group and 83 % (n = 33) in the control group (p = 0.240). The mean age was comparable between groups (50.9 ± 11.6 years in HypoPT vs 52.5 ± 10.6 years in controls; p = 0.591). Anthropometric measures including body mass index (BMI), waist, hip, wrist, and neck circumferences did not differ significantly between HypoPT patients and controls (BMI: 25.0 [22.8–28.4] vs 24.8 [21.7–26.1] kg/m^2^, p = 0.644; waist circumference: 87.5 ± 13.5 vs 85.6 ± 13.0 cm, p = 0.626). Biochemical analyses revealed significantly lower albumin-adjusted serum calcium levels in HypoPT patients compared to controls (8.54 ± 0.59 vs 9.5 ± 0.3 mg/dL; p < 0.001), alongside higher serum phosphate concentrations (4.11 ± 1.06 vs 3.26 ± 0.50 mg/dL; p = 0.0012), as expected. Serum PTH levels were reduced in the HypoPT group (median 12.15 [4.75–26.57] pg/mL) relative to controls (62.50 [39.50–77.15] pg/mL; p < 0.001). Vitamin D levels were similar between groups (32.5 ± 8.0 vs 29.7 ± 12.0 ng/mL; p = 0.316). Inflammatory markers showed higher erythrocyte sedimentation rate (ESR) in HypoPT patients compared to controls (30.0 [22.5–41.3] vs 24.0 [13.5–35.0] mm/h; p = 0.004), whereas C-reactive protein (CRP) levels and white blood cells did not differ significantly (0.19 [0.08–0.31] vs 0.11 [0.06–0.34] mg/dL; p = 0.292) and (5620 ± 1710 vs 5640 ± 1310 cells per microliter; p = 0.480). No significant differences were observed in fasting blood glucose (87.0 [85.0–101.0] vs 86.0 [80.8–92.5] mg/dL; p = 0.260) or thyroid-stimulating hormone (TSH) levels (1.70 [0.52–2.42] vs 1.24 [0.86–1.96] mIU/L; p = 0.934). In addition, no significant differences were observed in total cholesterol, HDL cholesterol and triglycerides (Table 1).

**Table 1:** Baseline characteristics of patients with hypoparathyroidism and healthy controls.

	Hyopoparathyroidism (n = 24)	Controls (n = 40)	P value
Sex, Female	96 % (n = 23)	83 % (n = 33)	0.240
Age, years	50.92 ± 11.61	52.5 ± 10.6	0.591
BMI, kg/m^2^	25.00 (22.84–28.43)	24.8 (21.7–26.1)	0.644
Waist circumference (cm)	87.53 ± 13.5	85.6 ± 13.0	0.626
Hip circumference (cm)	102.00 (96.00–110.00)	100.00 (94.00–104.25)	0.441
Wrist circumference (cm)	15.75 (14.75–16.00)	15.50 (15.00–16.00)	0.806
Neck circumference (cm)	34.00 (32.75–35.25)	33.00 (30.88–35.25)	0.563
Albumin-adjusted Calcium (mg/dL)	8.54 ± 0.59	9.5 ± 0.3	<0.001
Phosphate (mg/dL)	4.11 ± 1.06	3.26 ± 0.50	0,001
Vitamin D (ng/mL)	32.52 ± 8.00	29.65 ± 11.98	0.316
PTH (pg/ml)	12.15 (4.75–26.57)	62.50 (39.50–77.15)	<0.001
Creatinine (mg/dL)	0.87 ± 0.19	0.75 ± 0.12	0.001
Urinary calcium (mg/24h)	287.79 ± 17.22		
TSH (mlU/L)	1.70 (0.52–2.42)	1.24 (0.86–1.96)	0.934
White blood cells (cells per microliter)	5620 ± 1710	5640 ± 1310	0.480
ESR (mm/h^2^)	30.00 (22.50–41.25)	24.00 (13.50–35.00)	0.004
CRP (mg/dL)	0.19 (0.08–0.31)	0.11 (0.06–0.34)	0.292
Blood glucose (mg/dL)	87.00 (85.00–101.00)	86.00 (80.75–92.50)	0.260
Total cholesterol (mg/dL)	219.2 ± 53.85	186 ± 25.71	0.078
HDL cholesterol (mg/dL)	63.4 ± 18.88	63.38 ± 17.69	0.500
Triglycerides (mg/dL)	106.25 ± 40.92	89.37 ± 40.64	0.257
Disease duration, years	10.96 ± 8.33	–	–
Calcium supplements, mg/day	1408.33 ± 1069.71	–	–
Calcitriol, μg/day	0.70 ± 0.50	–	–
sNOX2-dp (pg/mL)	30.45 ± 14.19	17.11 ± 10.22	<0.001
H_2_O_2_ (μM)	81.33 ± 29.21	47.0 ± 24.13	<0.001
8-OHdG (ng/mL)	4.03 ± 2.31	1.55 ± 0.35	<0.001
PA (%)	46.67 ± 27.63	28.80 ± 31.9	<0.01
sP-selectin (ng/mL)	10.50 ± 4,14	4.44 ± 5.29	<0.001
sCD40L (ng/mL)	6.72 ± 2.01	2.37 ± 2.02	<0.001
vWF (ng/mL)	93.08 ± 16.05	75.68 ± 16.09	<0.001

Data are presented as mean ± standard deviation (SD) for normally distributed variables, median (interquartile range, IQR) for non-normally distributed variables, and percentage (n) for categorical variables. Abbreviations: BMI = Body mass index; PTH = Parathyroid hormone; ESR = Erythrocyte sedimentation rate; CRP=C-reactive protein; TSH = Thyroid-stimulating hormone.

**Fig. 1:**
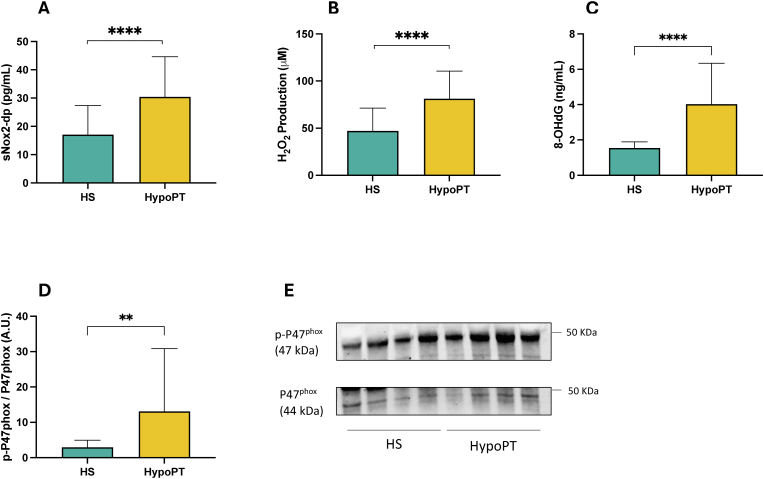
*In-vivo evaluation of oxidative stress in HypoPT and healthy subjects*. Serum levels of sNOX2-dp (A), H_2_O_2_ (B) and 8-OHdG (C) in 24 HypoPT subjects and 40 healthy subjects (HS) at basal conditions. P-p47^phox^/p47^phox^ expression in platelets from HS (N = 12) and HypoPT (N = 12) subjects at basal conditions and representative Western blot bands of p-P47^phox^/P47^phox^ (D–E). Differences between groups were analyzed by the Mann-Whitney test (for non-normally distributed data) or *t*-test (for normally distributed data). ∗∗p < 0.01; ∗∗∗∗p < 0.001.

Patients with HypoPT showed higher levels of sNOX2-dp (30.45 ± 14.19 vs 17.11 ± 10.22 pg/mL; p < 0.001), increased H_2_O_2_ production (81.33 ± 29.21 vs 47.0 ± 24.13 μM; p < 0.001) and higher serum concentrations of 8-OHdG (1.55 ± 0.35 vs 4.03 ± 2.31 ng/mL; p < 0.001), compared to controls, indicating increased oxidative stress (Table 1 and Fig. 1 panel A–C). In addition, we observed a significantly higher phosphorylation level of platelet p47^phox^ in HypoPT patients compared to controls, further supporting enhanced NOX2 activation in these patients (2.96 ± 1.99 vs 13.1 ± 17.7 A U.; p < 0.01) (Fig. 1 panel D and E). Similar results were observed for soluble P-selectin (sP-selectin) (10.50 ± 4.14 vs 4.44 ± 5.29 ng/mL; p < 0.001) and soluble CD40 ligand (sCD40L) (6.72 ± 2.01 vs 2.37 ± 2.02 ng/mL; p < 0.001). Moreover, we observed a significantly increased platelet aggregation percentage in patients with HypoPT compared to controls, (46.67 ± 27.63 vs 28.80 ± 31.9; p < 0.01). Finally, HypoPT patients also exhibited increased levels of vWF factor (93.08 ± 16.05 vs 75.68 ± 16.09 ng/mL; p < 0.001) compared to controls.

### 3.2 Ex vivo total thrombus-formation analysis system (T-TAS)

**Fig. 2:**
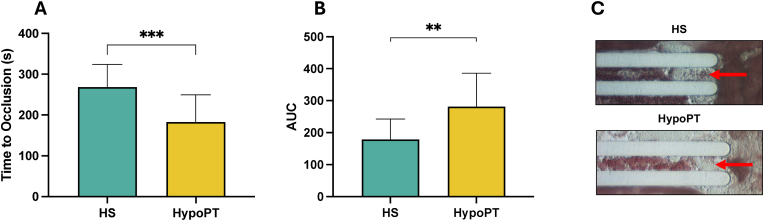
*Ex-vivo evaluation of thrombus formation in HypoPT and healthy subjects*. Time to occlusion (A) and AUC (B) from ex vivo total thrombus analysis formation (T-TAS) in 18 HypoPT subjects and 18 healthy subjects (HS). Representative image of thrombus formation (as indicated by the red arrows) in blood samples flowing through collagen-coated microcapillaries during the test (C). Data are reported as mean ± SD. ∗∗∗p < 0.001; ∗∗p < 0.01 were calculated using a parametric test (unpaired *t*-test).

In patients with HypoPT on conventional treatment we observed an accelerated thrombus growth as evidenced by reduced occlusion time and increased AUC compared to controls (p < 0.001 and p < 0.01 respectively) (Fig. 2A–C).

### 3.3 Oxidative stress and platelet activation in HypoPT patients treated with PTH

**Fig. 3:**
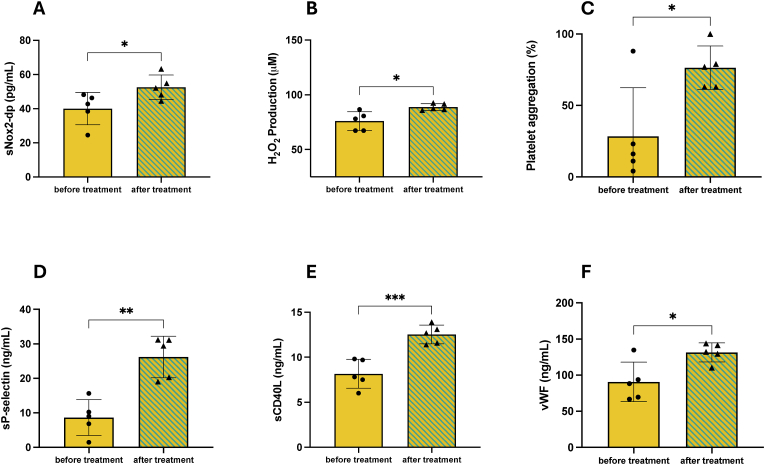
*In-vivo evaluation of oxidative stress and platelet activation in HypoPT enrolled subjects before and after* 24 months *of therapy with PTH (1–34).* Serum levels of sNOX2-dp (A) and H_2_O_2_ (B); platelet aggregation percentage (C); levels of plasma sP-selectin (D), plasma sCD40L (E); serum vWF (F) in HypoPT patients before and after *PTH (1–34)* therapy. (N = 5). Data are presented as mean ± SD in column graphs. Statistical significance between before and after treatment is indicated on the graphs (∗p < 0.05; ∗∗p < 0.01; ∗∗∗p < 0.001; paired *t*-test).

In HypoPT subjects, the treatment with PTH (1–34) determined high levels of sNOX2-dp and H_2_O_2_ production compared to pre-treatment status (p < 0.05) (Fig. 3A and B). Moreover, the PTH treatment induced a significant increase in ex vivo platelet aggregation (p < 0.05) (Fig. 3C). Similar results were observed for soluble s-Pselectin (p < 0.01) and sCD40L levels (p < 0.001) (Fig. 3D and E). Finally, HypoPT patients treated with PTH exhibited increased levels of vWF factor (p < 0.05) compared with pre-treatment conditions (Fig. 3F).

### 3.4 In vitro study

**Fig. 4:**
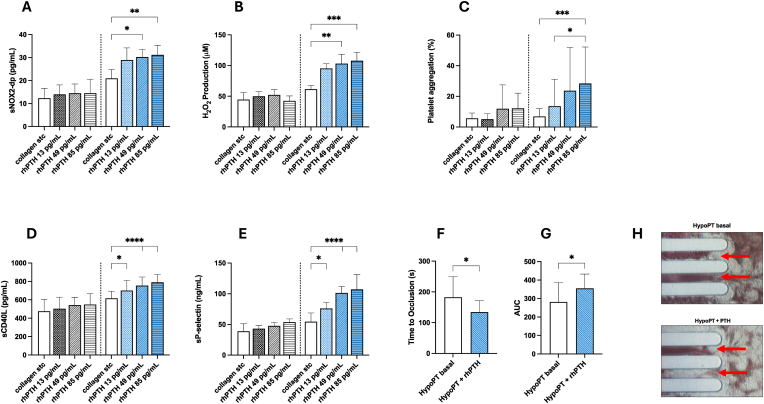
*Oxidative stress and platelet activation in HypoPT and controls after rhPTH (1–34) in-vitro administration.* Levels of sNOX2-dp (A); H_2_O_2_ production (B); platelet aggregation (C); sCD40L (D); sP-selectin (E) in platelets from HS and HypoPT after pre-incubation of PRP with growing concentrations (13 pg/mL; 49 pg/mL; 85 pg/mL) of rhPTH (1–34) and stimulated with collagen subthreshold. Data are reported as mean ± SD. ∗p < 0.05; ∗∗p < 0.01; ∗∗∗p < 0.001; ∗∗∗∗p < 0.0001 were calculated using a non-parametric test (Kruskal-Wallis). Time to occlusion (F) and AUC (G) of whole anticoagulated plasma of HypoPT (N = 18) before and after incubation with 49 pg/mL of rhPTH (1–34). Data are reported as mean ± SD ∗p < 0.05 using a parametric test (paired *t*-test). Representative image of white thrombus formation (as indicated by the red arrows) in blood samples flowing through collagen-coated microcapillaries during T-TAS analysis, in basal conditions and after the addition of 49 pg/mL of rhPTH (1–34) (H).

To evaluate the effects of exogenous PTH 1–34 on oxidative stress, platelet function and thrombus formation, we tested different concentrations of recombinant PTH (13; 49; 85 pg/mL) incubated with platelets isolated from controls and from HypoPT patients. The treatment of platelets from controls with PTH in the presence of STC of collagen did not affect oxidative stress (Fig. 4A and B), platelet function (Fig. 4C–E) and thrombus formation (Fig. 4F–H). On the other hand, PTH (13; 49; 85 pg/mL) with STC of collagen more potently increased oxidative stress (Fig. 4A and B), platelet function (Fig. 4C–E) and thrombus formation (Fig. 4F–H) in platelets from HypoPT patients.

In addition, we evaluated two key steps of NOX2-mediated platelet activation, such as PKC and P47 phosphorylation.

**Fig. 5:**
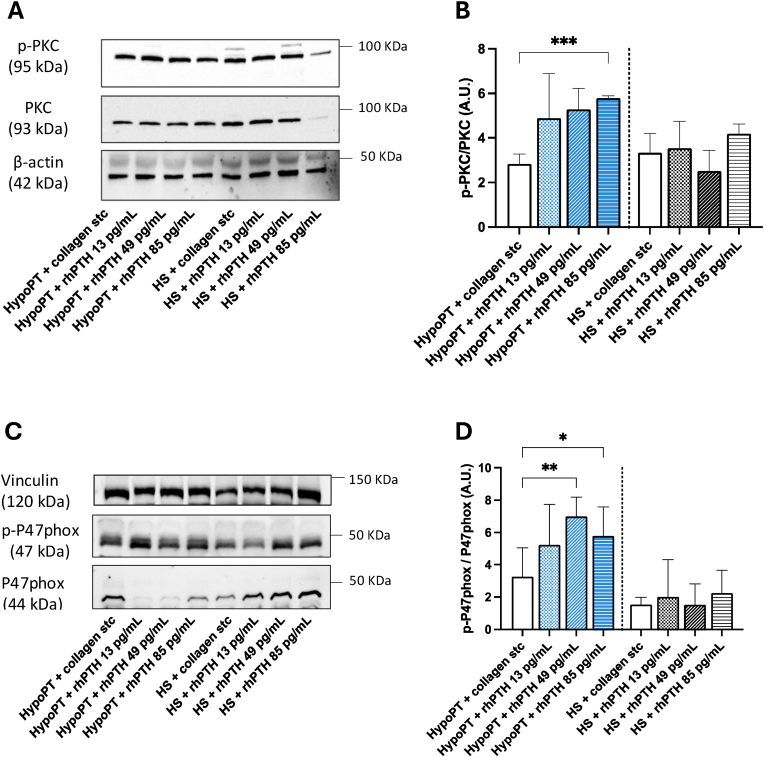
*Mechanism of oxidative stress activation in HypoPT and HS after rhPTH (1–34) in-vitro administration*. *p*-PKC and p-P47phox expression in platelets of HypoPT and HS subjects after incubation with increasing concentrations of rhPTH (1–34) (13 pg/mL; 49 pg/mL; 85 pg/mL) and stimulation with collagen subthreshold (0.25 μg/mL). Data are reported as mean ± SD ∗p < 0.05 ∗∗p < 0.01; ∗∗∗p < 0.001. Statistical significance was assessed by one-way ANOVA. Representative Western blot bands of *p*-PKC/PKC and p-P47phox/P47phox.

We observed that platelets from HypoPT patients treated with PTH (13; 49; 85 pg/ml) in the presence of STC of collagen showed an increased phosphorylation of PKC and P47phox compared to platelets stimulated with STC collagen alone (Fig. 5A–D). No significant changes were observed in platelets from HS (Fig. 5A–D).

**Fig. 6:**
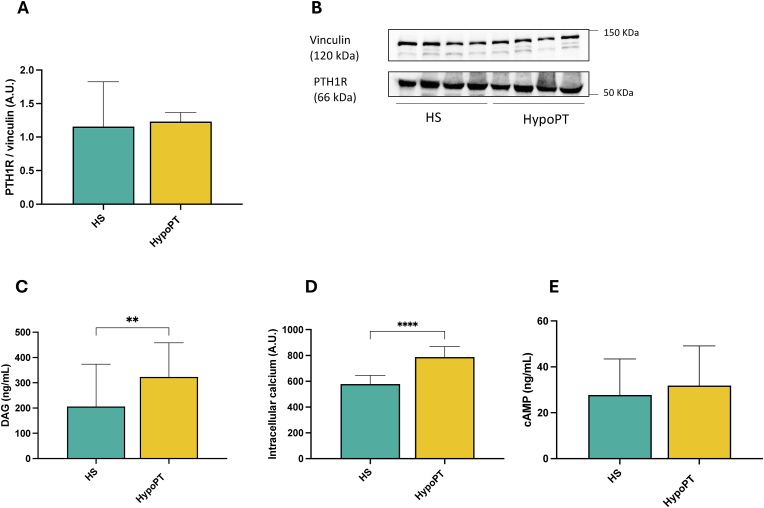
PTH1R expression in platelets from HS (N = 5) and HypoPT (N = 5) at basal conditions (A–B). Serum Diacylglycerol (DAG) concentrations (C) Intracellular calcium levels in platelets and (D) Serum circulating AMPc levels (E) in HS (N = 40) and HypoPT (N = 18) subjects at basal conditions ∗∗p < 0.01; ∗∗∗∗p < 0.001. Differences between groups were analyzed by Mann-Whitney test (for non-normally distributed data) or *t*-test (for normally distributed data).

To study the possible mechanism of PTH-mediated platelet activation, we investigated the PTH receptor (PTH1R) expression, which mediates pro-aggregating effects in response to both PTH and PTHrP, through a mechanism involving the activation of extracellular ligand-regulated kinases such as protein kinases. Our results showed that platelets from HS and HypoPT patients had a similar expression of the PTH1R (Fig. 6A and B). Considering this result and to explain the different effects of PTH in HS and HypoPT patients, we investigated the intracellular signaling pathway after PTH1R activation. Indeed, PTH bound to PTH1R activates a Gαs protein that results in the stimulation and propagation of the adenylyl cyclase/cAMP/protein kinase A (PKA) and a Gαq protein resulting in the activation of phospholipase Cβ/inositol trisphosphate (IP3)/Ca^2 +^/protein kinase C (PKC) pathways [27].

We found increased levels of intracellular calcium and diacylglycerol (DAG) in platelets from HypoPT patients compared to HS (Fig. 6C and D). Instead, no significant changes were observed in cAMP levels (Fig. 6E).

**Fig. 7:**
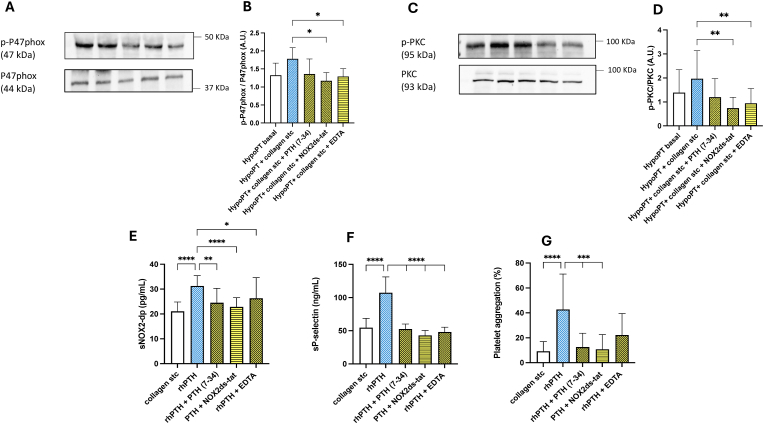
p-P47phox/P47phox (A–B) and *p*-PKC/PKC (C–D) expression in platelets from HypoPT patients pre-incubated with PTH (7–34) or NOX2-ds-tat (NOX2 docking sequence-tat) or EDTA and stimulated with collagen subthreshold. Levels of sNOX2-dp (E), sP-selectin (F) and platelet aggregation (G) from HypoPT patients' platelets after in vitro treatment with rhPTH alone and in combination with PTH (7–34) or NOX2-ds-tat or EDTA before collagen subthreshold stimulation. ∗p < 0.05; ∗∗p < 0.01; ∗∗∗p < 0.001; ∗∗∗∗p < 0.0001 were calculated using one-way ANOVA (for normally distributed data) and the Kruskal-Wallis test (for non-normally distributed data).

To confirm that the possible mechanism of PTH-mediated platelet activation was facilitated by PTH1R, intracellular Ca^2+^ mobilization and finally NOX2 activation, we incubated HypoPT platelets with PTH (7–34), that is a PTH/PTHrP receptor antagonist, NOX2-ds-tat, a specific inhibitor of NOX2 activation and EDTA as a calcium chelator. We observed that all inhibitors were able to reduce both PKC and P47 phosphorylation, NOX2 activation and platelet function (Fig. 7A–G).

## 4 Discussion

The results of the present study reveal that 1) postsurgical HypoPT has accelerated thrombus growth compared to healthy controls; 2) PTH (1–34) therapy in post-surgical HypoPT determined increased platelet aggregation and oxidative stress; 3) in vitro, platelets from HypoPT patients incubated with PTH showed increased oxidative stress, platelet activity and thrombus formation by a mechanism that involves PTH1R, intracellular calcium-mediated PKC phosphorylation and the pathway of NOX2 activation. This mechanism is mediated by NOX2 enzymatic activation through phosphorylation of its cytosolic subunit p47^phox^, a critical step for the assembly and activation of the NOX2 complex.

Data regarding the risk for CV morbidity and mortality in patients with postsurgical HypoPT are conflicting or derived mainly from registry studies or retrospective case-control studies. However, there is evidence showing an increase in the risk of developing cardiovascular diseases in postsurgical chronic HypoPT patients. Indeed, according to the results of a recent meta-analysis, postsurgical chronic HypoPT increased the risk of developing cardiac morbidities, comprises ischemic heart disease and strokes. The risk of cardiac problems in HypoPT patients doubles that of the control group [28]. In addition, a recent study confirmed an increased risk of multiple cardiovascular outcomes in patients with chronic HypoPT, particularly among women, including valvular heart disease, peripheral artery disease, heart failure, atrial fibrillation, myocardial infarction, and cardiovascular mortality [6].

Following these observations, we have recently demonstrated that HypoPT have impaired platelet reactivity and endothelial activation [15], both processes unquestionably involved in the pathogenesis of atherothrombosis. In this study, we confirm and extend the previous data also showing an increased risk of atherothrombosis in these patients as indicated by significant differences in time to occlusion and AUC at T-TAS analysis, both parameters of increased risk, compared to control subjects.

These findings confirm an alteration in platelet function in patients with HypoPT and highlight the role of PTH in regulating platelet activity. We further investigated the impact of PTH 1–34 therapy on platelet function and activation, assessing oxidative stress and platelet reactivity in a subgroup of subjects before and after 24 months of PTH 1–34 treatment. Our results demonstrate that, in these patients, PTH (1–34) is associated with a further increase in oxidative stress and platelet reactivity. Although these preliminary findings are based on a small patient cohort, they point to the importance of further investigations, especially in light of the recent advances in PTH replacement therapies and the growing number of patients likely to receive such treatment. However, clinical studies conducted to date have not demonstrated an increased thrombotic risk associated with PTH replacement therapy.

To explore the mechanisms underlying the enhanced platelet activation observed in HypoPT patients treated with PTH analogues, we conducted an in vitro study assessing the effects of rhPTH (1–34) on oxidative stress, platelet function, and thrombus formation. The impact of PTH on platelet activation remains controversial. Earlier studies reported that PTH inhibits platelet aggregation induced by various agonists (e.g., ADP, collagen, thrombin) [29], whereas more recent evidence suggests a pro-thrombotic role for PTH. In particular, elevated PTH levels have been positively associated with platelet indices in women with symptomatic heart failure [30], and PTHrP, by interacting with PTH1R on human platelets was shown to potentiate platelet aggregation in response to different stimuli [13].

In our study, we found that rhPTH (1–34) significantly enhanced oxidative stress, platelet activation, and thrombus formation only in platelets isolated from HypoPT patients, particularly when stimulated with a STC of collagen. This pro-aggregating effect was not observed in platelets from healthy subjects, suggesting a disease-specific susceptibility to PTH-mediated activation. In particular, the lack of response in healthy platelets indicates that the presence of HypoPT-associated alterations, such as chronic PTH deficiency, altered calcium-phosphate homeostasis, or adaptive changes in intracellular signaling, may prime platelets for enhanced responsiveness to exogenous PTH. These findings reinforce the notion that platelet hyperreactivity to rhPTH is not an intrinsic property of the hormone itself but rather emerges in the context of a specific pathophysiological background.

Mechanistically, our data indicate that rhPTH increases phosphorylation of PKC and P47 in HypoPT platelets. Since phosphorylation of P47 is essential for the activation of the NADPH oxidase isoform NOX2, and PKC is a well-known upstream activator of this process, our results point toward a functional PKC–P47phox–NOX2 axis as a key mediator of PTH-induced platelet activation in HypoPT. This is further supported by our observation that inhibition of PTH1R (with PTH (7–34), chelation of intracellular Ca^2+^ (with EDTA), and direct inhibition of NOX2 (with NOX2-ds-tat) all attenuated 10.13039/100012258PKC and P47 phosphorylation, reduced NOX2 activation, and normalized platelet function.

Interestingly, the expression of PTH1R was comparable in both HS and HypoPT subjects, suggesting that the differential response is not due to receptor overexpression but rather to alterations in downstream signaling pathways. Accordingly, we observed increased intracellular calcium and DAG levels in HypoPT platelets treated with PTH, while cAMP levels remained unchanged. These findings imply a predominant activation of the Gαq/PLCβ/IP3/Ca^2+^/PKC pathway, rather than the Gαs/cAMP/PKA axis, in mediating the pro-aggregatory effects of PTH in this context [31].

Taken together, our results provide novel mechanistic insights into the enhanced platelet activation related to rhPTH administration in HypoPT patients and highlight a disease-specific sensitivity of platelets to PTH-mediated activation through the PKC-NOX2 pathway. This might have important clinical implications for the management of cardiovascular risk in HypoPT individuals undergoing PTH replacement therapy.

Our findings confirm that the impaired platelet reactivity and increased oxidative stress result in enhanced thrombus formation process and may represent clinically relevant features of hypoparathyroidism, potentially contributing to the increased cardiovascular risk observed in these patients. Furthermore, the observation that PTH (1–34) therapy may exacerbate these alterations highlights the need for future research for personalized cardiovascular evaluation. A major strength of our study lies in its novelty and these findings open new avenues for investigating the systemic effects of PTH and its role beyond mineral metabolism. The main limitations of this study include the small sample size of the longitudinal subgroup treated with PTH (1–34), which may limit the generalizability of our findings. In addition, the observational design does not allow us to establish causal relationships. In addition, a limitation of our study is the underrepresentation of male subjects within our patient cohort. Given the predominance of female participants, the generalizability of our findings to the male population is limited. Therefore, future studies specifically addressing platelet function and cardiovascular risk in male HypoPT patients are warranted to determine whether similar mechanisms and risks are present across sexes.

Moreover, further mechanistic insights are warranted to validate and expand upon these preliminary findings.

In conclusion, our findings confirm that HypoPT subjects exhibit enhanced platelet activity compared to controls, and PTH (1–34) might accelerate this condition. In particular, in HypoPT, PTH was able to induce platelet activation by a mechanism that involved PTH1R and the pathway of NOX2 activation that includes the intracellular calcium-mediated PKC and P47 phosphorylation (see graphical abstract).

## CRediT authorship contribution statement

**Alessandra D'Amico:** Conceptualization, Data curation, Formal analysis, Funding acquisition, Writing – original draft. **Gaia Tabacco:** Conceptualization. **Cristina Nocella:** Conceptualization. **Anda Mihaela Naciu:** Writing – original draft. **Annunziata Nusca:** Methodology. **Francesco Piccirillo:** Methodology. **Michele Mattia Viscusi:** Methodology. **Federico Bernardini:** Methodology. **Valentina Valenti:** Methodology. **Angela Leonardo:** Methodology. **Sebastiano Sciarretta:** Writing – review & editing. **Ernesto Greco:** Writing – review & editing. **Gianmarco Sarto:** Methodology. **Beatrice Simeone:** Methodology. **Luca D'Ambrosio:** Methodology. **Giacomo Frati:** Writing – review & editing. **Athanasios D. Anastasilakis:** Methodology. **Francesco Grigioni:** Writing – review & editing. **Nicola Napoli:** Methodology. **Maurizio Forte:** Supervision. **Andrea Palermo:** Supervision. **Roberto Carnevale:** Supervision.

## Funding

This work was supported by the European Union - Next Generation EU- NRRP M6C2 - Investment 2.1 Enhancement and strengthening of biomedical research in the NHS; (PNRR-MAD-2022-12376779; CUP: F23C220010900006) (to R.C. and M.F.).

## Declaration of competing interest

The authors declare that they have no known competing financial interests or personal relationships that could have appeared to influence the work reported in this paper.

## Data Availability

Some or all datasets generated during and/or analyzed during the current study are not publicly available but are available from the corresponding author on reasonable request.
